# Finite element modeling of effects of tissue property variation on human optic nerve tethering during adduction

**DOI:** 10.1038/s41598-022-22899-2

**Published:** 2022-11-08

**Authors:** Joseph Park, Andrew Shin, Joseph L. Demer

**Affiliations:** 1 Department of Ophthalmology, Stein Eye Institute, University of California, Los Angeles, Los Angeles, CA 90095-7002 USA; 2 Department of Bioengineering, University of California, Los Angeles, Los Angeles, CA USA; 3 Intelon Optics Inc., Lexington, MA USA; 4 Neuroscience Interdepartmental Program, University of California, Los Angeles, Los Angeles, CA USA; 5 Department of Neurology, University of California, Los Angeles, Los Angeles, CA USA

**Keywords:** Computational models, Visual system, Physiology

## Abstract

Tractional tethering by the optic nerve (ON) on the eye as it rotates towards the midline in adduction is a significant ocular mechanical load and has been suggested as a cause of ON damage induced by repetitive eye movements. We designed an ocular finite element model (FEM) simulating 6° incremental adduction beyond the initial configuration of 26° adduction that is the observed threshold for ON tethering. This FEM permitted sensitivity analysis of ON tethering using observed material property variations in measured hyperelasticity of the anterior, equatorial, posterior, and peripapillary sclera; and the ON and its sheath. The FEM predicted that adduction beyond the initiation of ON tethering concentrates stress and strain on the temporal side of the optic disc and peripapillary sclera, the ON sheath junction with the sclera, and retrolaminar ON neural tissue. However, some unfavorable combinations of tissue properties within the published ranges imposed higher stresses in these regions. With the least favorable combinations of tissue properties, adduction tethering was predicted to stress the ON junction and peripapillary sclera more than extreme conditions of intraocular and intracranial pressure. These simulations support the concept that ON tethering in adduction could induce mechanical stresses that might contribute to ON damage.

## Introduction

Recent magnetic resonance imaging (MRI) has suggested that the optic nerve (ON) tethers the globe in adduction, which is rotation towards the midline^1,2^. During adduction beyond the 26° threshold for tethering^3^, contraction of the medial rectus muscle imposes torque on the globe that is largely opposed by reaction force in the ON whose proximal end is fixed at the apex of the eye socket^1,2^. Thus during tethering, the ON represents a significant mechanical load on the eyeball and extraocular muscles^1^.

Eye movements large enough to cause ON tethering are common in daily life. During reaching, people make rapid eye movements called saccades up to 40°–45°^4^, and about three saccades of various sizes are made every second^5^ totaling over 180,000 daily^6^. Eye movements especially apt to cause ON tethering occur when we actively move our heads^7^ and while we walk or run. When moving this way, people may make 25°–45° saccades^7^. When we turn our heads to look, eye movements average around 30°^8,9^. Tethering of the ON is therefore ubiquitous in healthy people, even more so in people with crossed eyes, in whom ON damage attributed to glaucoma occurs oftener than in people with straight eyes^10^.

Optical imaging in living people shows that during adduction there is a deformation of the optic disc, a structure that is the anterior termination of the ON at its junction with the eyeball, and surrounding tissues are also deformed^3,11^. Such deformations of the disc and nearby tissues^3,11^ exceed many-fold those resulting from extremely high intraocular pressure (IOP)^12^ in the range potentially damaging the retina^13^. Oculorotary forces may even cause bleeding from vessels around the disc^14^. In healthy people, MRI reveals that the ON stretches during adduction^15^, while in patients with open angle glaucoma at normal IOP, failure of the ON to stretch is associated with globe retraction that does not occur in healthy people^1^. In highly nearsighted eyes, ON tethering in adduction causes significant retraction of the eyeball into its socket^1^. Adduction is exaggerated in people with esotropic strabismus, which in as Korean population study was found to be a much stronger risk factor for primary open angle glaucoma (POAG) than elevated IOP^16^. These observations suggest that adduction tethering is very frequent.

The phenomenon of adduction tethering is particularly relevant because of the accumulating evidence that elevated IOP is not the cause of ON damage in primary open angle glaucoma, even though medical or surgical IOP reduction remains the objective of all current forms of glaucoma treatment, and despite common adverse effects of such treatment. For example, in the seminal Ocular Hypertension Treatment Study, IOP reduction decreased the incidence of POAG by 5.1%, but increased the relative risk of cataract by 49%^17^. But most patients with POAG, especially in Asia^18–22^, have never experienced abnormally high IOP^23^ and but develop ON damage at normal (< 22 mmHg) IOP^24^. IOP is normal in POAG in 30–39% of whites^25–27^, 57% of blacks^28^, 70% of Chinese^22^, 92% of Japanese^19^, and 99.4% of Koreans^10^. Multiple studies have failed to establish statistical relationship of IOP to POAG^22,29^ or to progressive ON damage^30–32^. Even when reduced by treatment, IOP level does not statistically predict progressive ON damage^31,33^, and around 20% of patients develop such damage 5 years after 30% IOP reduction from levels already statistically normal^24^, even when extremely low IOP itself begins to cause visual loss^34^. Clearly, other sources of stress on the ON besides IOP deserve consideration. We^1–3,11,35–37^ and others^16,38–40^ have suggested that eye movements could constitute another important source of mechanical damage to the ON.

With the forgoing in mind, ocular deformations during horizontal eye rotations have been investigated in early finite element modeling (FEM) studies^38,41,42^. Shin et al. modeled tethering by a straight ON but employed bovine tissue properties and made several anatomical simplifications^41^. Wang et al. simulated small angle eye rotation from the central gaze when the ON path remained sinuous rather than straightened by tethering^38,42^, predicting ON deformation by abduction and adduction to angles less than 26°. Although these studies have provided insights, these studies do not clarify the mechanical effects of ON tethering in humans.

We have published data showing that hyperelastic properties of human tissues relevant to this simulation vary substantially, both among individuals, and regionally within the same eyes^43^. The current FEM investigates the effects of these property variations on local tissue loadings during adduction tethering. We hypothesize in the current study that combinations of local biomechanical properties influence stress and strain distributions during adduction tethering, and that FEM employing the observed range of variation in tissue properties may suggest combinations potentially causal to pathological loading, recognizing that their nonlinearity implies that considering average tissue behavior does not necessarily describe the average mechanical behavior^44^. We here report the interaction of adduction tethering with changes in IOP and intracranial pressure (ICP). The present investigation employs average anatomical dimensions for the eye and orbit, since the simultaneous variation of both anatomy and material properties, as two orthogonal parameter spaces, would prohibitively increase the complexity of the results. Effects of anatomical variations will be reserved for future description.

## Results

### Model implementing average tissue properties

**Figure 1:**
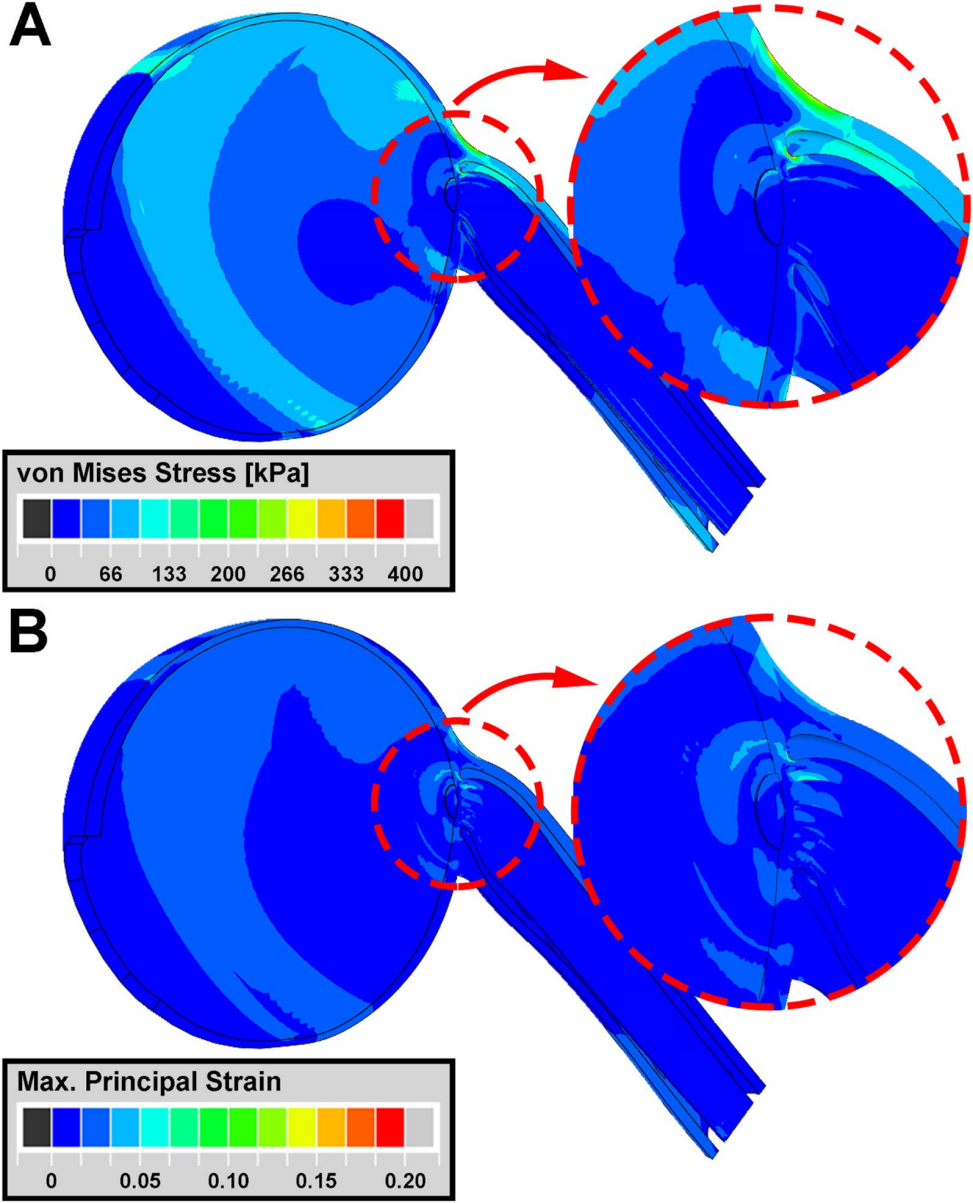
Simulation of adduction to 32° from initial ON tethering at 26°, employing average measured tissue hyperelastic functions. Heat maps of (**A**) von Mises stress and (**B**) principal strain. Stress–strain effects mainly occur in and around the ON head in the region enclosed by red dotted circles.

Stress and strain during adduction 6° beyond the 26° threshold of tethering are illustrated in Fig. 1 assuming average tissue properties (Table 1)^43^. For this 6° incremental adduction, tractional ON loading propagated from the temporal ON junction to the inner peripapillary sclera, which experienced around 140 kPa stress and 5% strain, with lesser deformations widely distributed throughout the sclera and ON sheath (Fig. 1). There was 4% strain in the ON neural tissue at the temporal border of the lamina cribrosa (LC), the perforated connective tissue structure in the posterior optic disc through which the axons of the ON pass as they exit the eye to join the ON.

**Table 1:** Hyper-elasticity: reduced polynomial model and linear elasticity (MPa).

Region	Stiffness	C10	C20	C30 (× 10^3^)	C40 (× 10^3^)	C50 (× 10^3^)	C60 (× 10^3^)	D1	D2–D6
Anterior sclera	Average	0.926	511	− 42.6	2260	− 59,300	594,000	0.044	0
Equatorial sclera	Average	0.390	252	− 11.2	219	–	–	0.104	
Posterior sclera	Average	0.633	144	− 6.08	153	− 1930	9550	0.064	
	Stiff	1.056	179	− 7.31	179	− 2190	10,300	0.038	
	Compliant	0.069	46	− 7.4	4.16	–	–	0.587	
Peripapillary sclera	Average	0.170	44	− 1.43	27.59	− 250	845	0.238	
	Stiff	0.060	48	− 0.412	1.95	− 4300	3510	0.674	
	Compliant	0.080	− 0.539	0.00881	− 0.0198	15,100	–	0.400	
Optic nerve (ON) sheath	Average	0.492	91	− 1.78	16.3	–	–	0.083	
	Stiff	0.221	2870	− 11.5	280	− 3310	14,700	0.184	
	Compliant	0.127	25	− 0.446	2.92	–	–	0.318	
Lamina cribrosa	Average	0.281	16	− 0.0445	0.05	–	–	0.072	
ON connective tissue	Average	0.626	98	− 3.48	70.2	− 679	2490	0.065	
	Stiff	1.226	126	− 1.78	8.92	–	–	0	
	Compliant	0.160	21.6	− 0.311	1.81	–	–	0	

Region	Stiffness	Young’s modulus	Poisson’s ratio						
ON neural tissue	Average	0.001195	0.48						

### Sensitivity analysis

**Figure 2:**
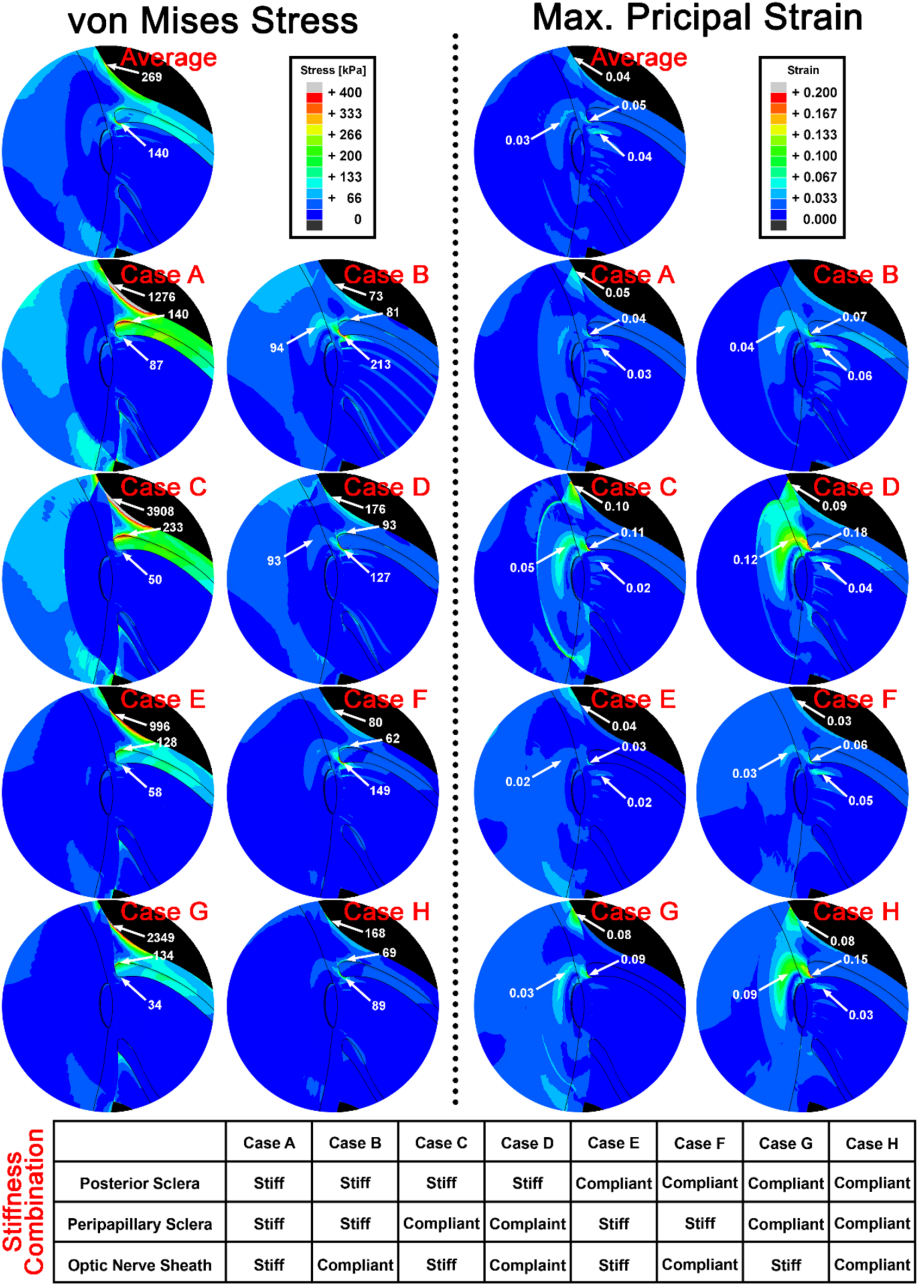
Sensitivity to variations in regional ocular material properties, as implemented by reduced polynomial functions for which the indicated qualities are shorthand, during adduction 6° beyond onset of tethering at 26°. Stiff and compliant material properties were set to 95th and 5th percentile values, respectively, of stress–strain functions measured after preconditioning^43^ (details in Table 1). Material properties for regions not noted here were set to average observed reduced polynomial functions.

Systematically modeling stiff versus compliant material properties in local regions (Table 1) predicted large variations in stress and strain during adduction tethering (Fig. 2). We selected posterior, peripapillary sclera, and ON sheath, as tissues of interest likely to influence the deformation of the optic disc during adduction, and performed multiple simulations assuming various combinations of local tissue properties in those regions as described in tabular entries of Fig. 2.

Case A assumed stiff tissue properties in the posterior and peripapillary sclera, as well as the ON sheath (Fig. 2); with this combination, stress was low at 87 kPa in the temporal aspect of the optic disc, but high at 1276 kPa at the junction of the ON sheath with the posterior sclera. The diffusely stiff connective tissues thus reduced stress on the ON at the disc. Case A showed similar or slightly less strain than the average case throughout the regions evaluated. However, when the ON sheath was compliant but posterior and peripapillary sclera were stiff in Case B, stress in the temporal aspect of the optic disc and peripapillary sclera was increased to 213 kPa and 94 kPa, respectively; this suggests that stress is transferred to the vulnerable optic disc when the ON sheath is compliant. Strain in the optic disc, peripapillary sclera, and neural tissue were twice those in case A. Strain in retrolaminar neural tissue was 6% in Case B, the greatest of all cases modeled.

Case C that assumed stiff posterior sclera and ON sheath, with compliant peripapillary sclera, showed the most extreme stress concentration in the junction of ON sheath and posterior sclera at 3908 kPa, although stresses in peripapillary sclera and the ON were low. Because the peripapillary sclera was the only complaint tissue, strain was greatest there.

Assuming stiff posterior sclera but compliant ON sheath and peripapillary sclera in case D, the stress was moderate in all regions, but the strains on the temporal peripapillary sclera and optic disc were 12% and 18%, respectively. These were the highest strains in these regions among all cases modeled.

It is informative to consider the effect of posterior scleral properties by contrasting Cases A, B, C, and D that have stiff posterior sclera, with Cases E, F, G, and H that have compliant posterior sclera. The four instances in each group systematically vary the possible combinations of stiffness in the peripapillary sclera and ON sheath in respective order (Fig. 2). This provides the following contrasting case pairs for visual inspection in Fig. 2: A–E, B–F, C–G, and D–H. Consideration of these pairs suggest that in cases E, F, G and H, the compliant posterior sclera absorbs deforming force, leading to lesser stresses in the other tissues. For example, in Case C with stiff posterior sclera there was 3908 kPa stress at the junction of the ON sheath and the posterior sclera, but for Case G with compliant posterior sclera this stress was 40% lower at 2349 kPa.

The selection of paired extremes of ON sheath properties (Cases A–B, C–D, E–F, G–H) indicated that when the ON sheath was relatively compliant, stresses in the posterior sclera and ON sheath were smaller, but stresses in the peripapillary sclera, and ON junction were larger. Strains during adduction tethering in the retrolaminar ON neural tissue, peripapillary sclera, and ON junction were greater with a compliant than stiff ON sheath.

Comparison of contrasting paired extremes of peripapillary scleral properties (Cases A–C, B–D, E–G, F–H) demonstrated that stiff peripapillary sclera reduced stress in the ON sheath, but increased it on the ON junction. Stiff peripapillary sclera experienced smaller strains within it but allowed larger strains in ON neural tissue.

Cases such as A, C, D, E, G, and H predicted less than average case stresses on the temporal optic disc, while cases B and F exhibited more than the average case. All except cases A and E exhibited higher strains than for the average case in the temporal optic disc.

### Scleral displacement

**Figure 3:**
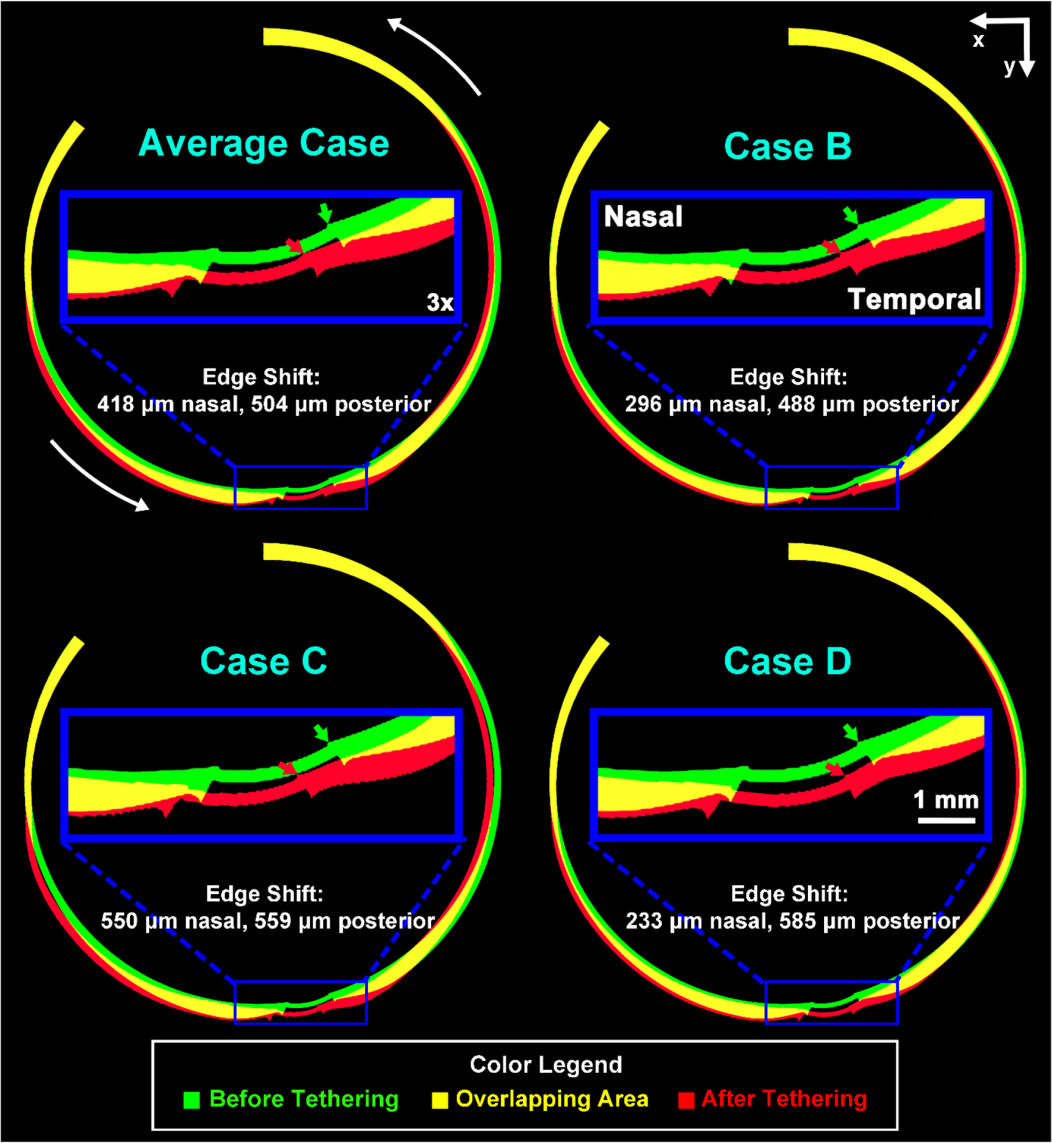
Horizontal cross sections of finite element model superimposing initial configuration of 26° adduction at initiation of tethering (green) with final configuration of 32° adduction (red) for various combinations of observed tissue properties as defined in Fig. 2. Yellow region represents overlap. Blue inset shows 3 × magnified view of the optic disc region where arrows indicate locations of temporal edge of the lamina cribrosa, whose displacements are listed numerically for each case.

Scleral displacement during adduction tethering was visualized by superimposing the model’s rigid anterior sclera in initial and tethered adducted positions (Fig. 3). In the simulation employing average tissue properties, the temporal LC edge shifted 418 μm nasally and 504 μm posteriorly during adduction tethering. Extreme case B had smaller LC displacement in both nasal-temporal (296 μm nasal) and anterior–posterior (488 μm posterior) directions than the average case (Fig. 3). Extreme cases C and D exhibited larger LC displacement nasally and posteriorly than the average case: there was 550 μm nasal and 559 μm posterior shift in case C, and 233 μm and 585 μm shift in case D, respectively.

### Pressure

**Figure 4:**
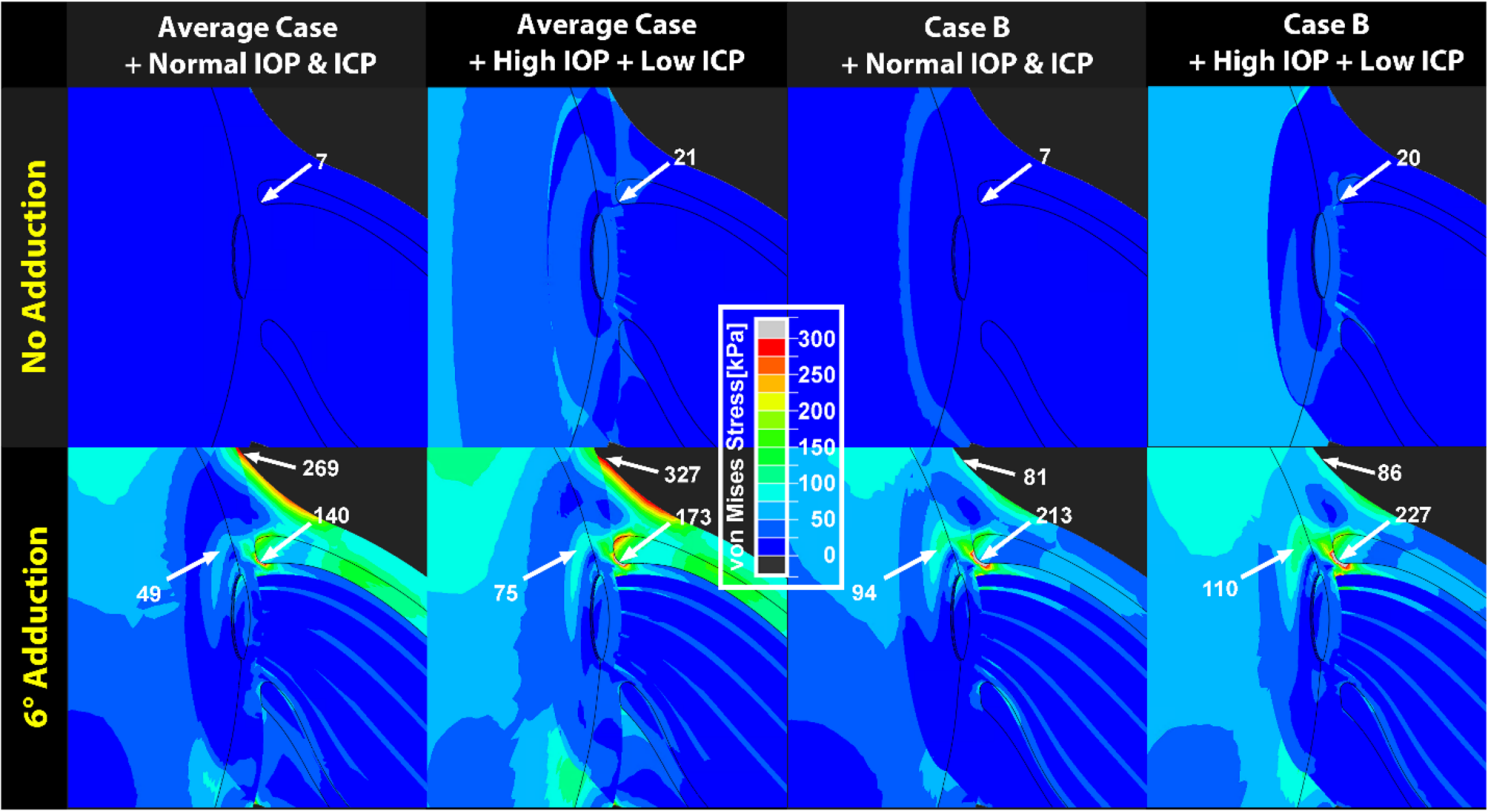
Finite element model of adduction 6° beyond onset of tethering at 26° demonstrating additional influences of intraocular pressure (IOP) and intracranial pressure (ICP) on stress distributions in the peripapillary and optic disc region. Material property cases are defined in Fig. 2. In upper panels without adduction tethering, note small stress due to 40 mmHg IOP (high) and 4 mmHg ICP (low), in comparison with lower panels showing larger effect of adduction. Comparison of lower right two panels for Case B demonstrates that during adduction, stress in the temporal peripapillary sclera and ON junction is 14 kPa higher when IOP is high and ICP is low, than when these pressures are normal.

The normal range of IOP for the healthy young adult is 7–21 mmHg^45^ and average IOP is about 15 mmHg. IOP exceeding 21 mmHg is often regarded as potentially pathological to the ON, and may eventually result in ON damage termed glaucoma^46^. Hydrostatic pressure imposes mechanical loading on the eye, and in particular the pressure differential across the LC between IOP and ICP has been proposed as damaging to the ON in glaucoma^12,47,48^. To compare the effect of adduction tethering with that of translaminar pressure, we simulated adduction tethering along with extreme 36 mmHg pressure gradient caused by high (40 mmHg) IOP and low ICP (4 mmHg)^49^. As shown in Fig. 4, for the case of average tissue properties, stress in the temporal ON junction with extreme translaminar gradient during adduction tethering (173 kPa) modestly exceeded stress during tethering at normal pressures (140 kPa). During adduction in case B, there was 213 kPa stress with normal IOP at the temporal ON junction, increasing slightly to 227 kPa with extreme translaminar gradient as a roughly additive effect.

### Stiff optic nerve concentrates stress

**Figure 5:**
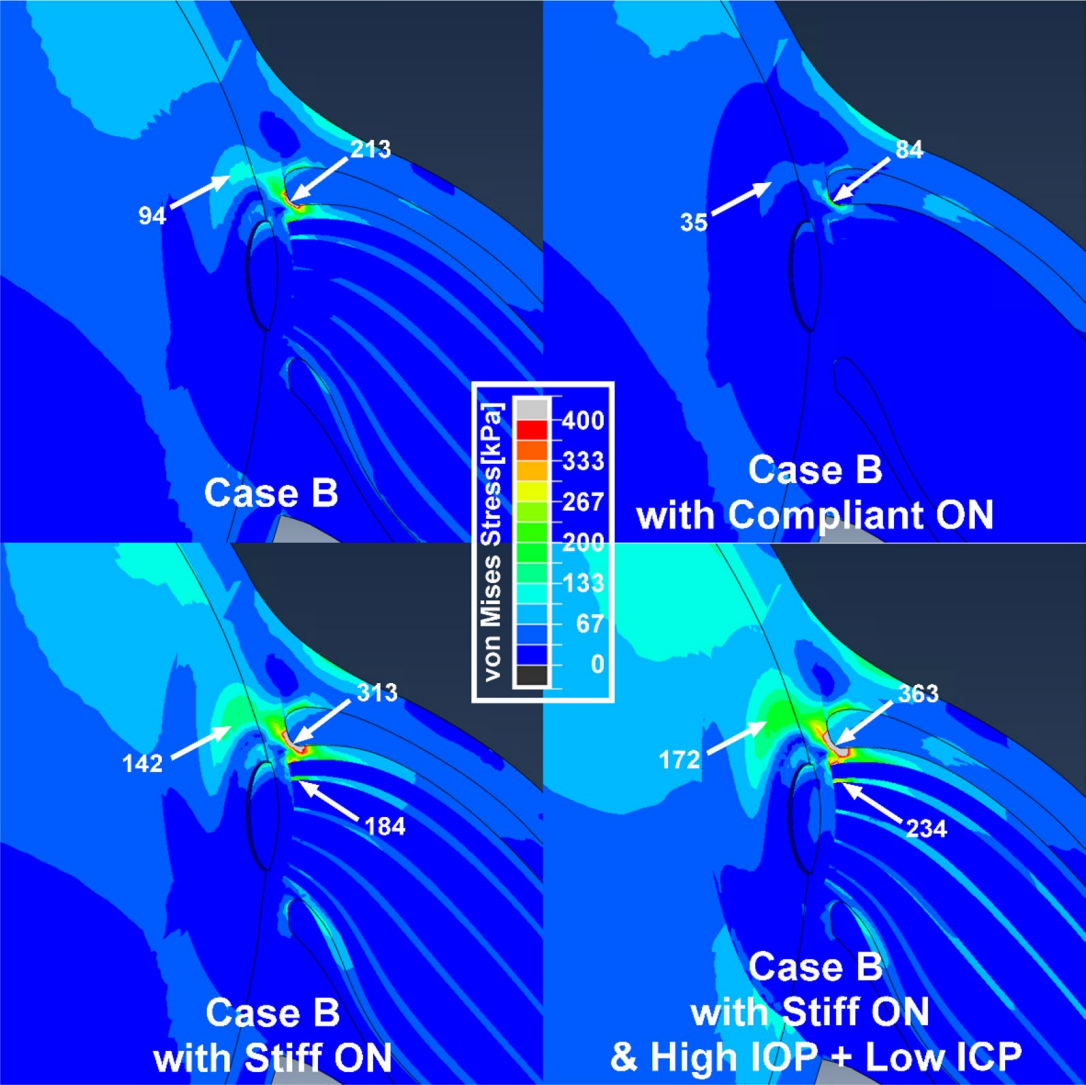
Sensitivity to ON stiffness greater or less than average, assuming the least favorable combination of other tissue properties (case B). Simulations assumed normal IOP and ICP, except for the right bottom panel that assumes an extreme translaminar pressure gradient (40 mmHg IOP and 4 mmHg ICP).

Sensitivity to variation in ON properties was simulated assuming the stiff posterior and peripapillary tissue properties of Case B (Fig. 5). Simulation that assumed normal IOP and ICP indicated greater stress in and around the temporal ON junction when the ON was stiffer (Fig. 5, lower left) than average (Fig. 5, upper left), but less stress when the ON was less stiff (Fig. 5, upper right) than average (Fig. 5, upper left). Stress on the optic disc in case B assuming normal ON stiffness (213 kPa, Fig. 5, upper left) was about 32% less than that for case B with a stiff ON (313 kPa, Fig. 5, lower left), but was much less when the ON was relatively compliant (84 kPa, Fig. 5, upper right). The effect of an extreme translaminar pressure gradient was simulated with a stiff ON (Fig. 5, lower right), which increased stresses on the temporal peripapillary sclera (172 kPa), intrinsic ON connective tissue (234 kPa), and ON junction (363 kPa) to more than 70% above the case with average tissue properties.

## Discussion

When ocular adduction reaches an angle that exhausts ON redundancy so that the ON itself becomes a tether, reaction force to the contracting medial rectus muscle is applied to the eyeball by tension in the ON and its sheath. The present FEM suggests that ON tethering during adduction results in widespread loading of the globe and ON depending on variations in ocular tissue properties within the experimentally measured range. By examining combinations of observed tissue mechanical properties, we explored some bounds on possible local loadings caused by ON tethering in adduction. This FEM extends the analysis of adduction tethering from bovine^41^ parameterization to more relevant, nonlinear human tissue properties^43^. Although these simulations cannot determine if simulated loadings during adduction tethering could induce biological effects such as peripapillary atrophy or glaucomatous ON damage, the stresses and strains predicted by this FEM of certain tissue properties are considerably higher than those predicted for IOP elevation that is clearly regarded as pathological in glaucoma. Mechanical effects of IOP appear roughly additive to those of ON tethering.

The most significant insight emerging from this FEM is that combinations of variations within the measured range of tissue properties markedly influence magnitudes and distributions of resulting stress and strain on the ON and posterior eye during adduction tethering. In extremes, adduction tethering is predicted to concentrate large stresses (Case B) and strains (Cases C and D) in peripapillary sclera and the optic disc region where peripapillary atrophy typically develops^50–54^, and where optic neuropathy typically occurs in primary open angle glaucoma^47,55,56^. The 2–6% of strain range in the ON junction shown in this FEM is comparable to the 1.8–4.1% strain around the LC and ON neural tissue predicted by Wang et al.’s FEM^38,42^ although Wang investigated small 13° adduction that does not cause ON tethering.

Tensile testing has shown only modest cross-correlation among mechanical properties of various regions of the globe, ON, and sheath in individual eyes^43^. For example, the strongest regional correlation demonstrated that only 59% of the variation in ON sheath tangent modulus is predictable from variation in anterior scleral modulus, and the lowest regional correlation showed that as little as 1% of the variation in ON modulus is predictable from its sheath modulus. This justifies independently varying stiffnesses of each ocular region in sensitivity analysis of Fig. 2.

The normal ON stretches harmlessly during eye rotations^15^, so that strain within physiologic limits must generally not be pathological, but presumably acts as a buffer to reduce stress at the junction of the ON and eyeball. Therefore, we suggest that adduction-related stress rather than strain may be a pathologic factor. Specific downstream mechanobiological mechanisms that might mediate stress effects are implicit or explicit in most theories of glaucoma^13,57–60^.

Focal stress concentrated on the ON connective tissue is believed to induce tissue remodeling^13,56,58–64^. The FEM with average tissue properties (Fig. 1) indicated concentration of moderate stress and strain during adduction tethering in and around the optic disc, and the ON and its sheath. Both the temporal and nasal sides of the peripapillary sclera and LC shift nasally and posteriorly during adduction tethering (Fig. 3). Stress and strain concentrations are predicted to be greater in the temporal than nasal disk (Fig. 1), associated with nasal and posterior LC movements^65,66^. Figure 2 suggests that stiff posterior sclera induces both greater stress and strain on the peripapillary sclera, ON, and ON sheath by transmitting stress to the posterior region rather than absorbing it by posterior scleral deformation, which buffers stress and strain to protect the optic disc^67^.

Stiff peripapillary sclera (Cases A, B, E, F) reduces ON sheath stress and limits its overall strain at the cost of increasing stress in and around the temporal peripapillary sclera and ON junction. Interestingly, compliant peripapillary sclera relieves ON stress (Case D in Fig. 2). Low ON sheath stiffness (Cases B, D, F, H) is associated with greater stress near the ON junction and greater strain on retrolaminar neural tissue during adduction tethering, effects that might plausibly induce optic neuropathy when repeated sufficiently^47,56^. The most favorable combination (Case G) of material properties to minimize temporal ON junction stress during adduction tethering maybe when compliant posterior and peripapillary sclera absorb the force of adduction tethering, but a stiff ON sheath protects the compliant ON from loading. The least favorable combination (Case B) of material properties heavily stressing the temporal ON junction may be when stiff posterior and peripapillary sclera transfer adduction force to an ON junction that is not well protected by the ON sheath. This combination of factors might, after sufficient repetition of eye movements over a lifetime, lead to ON pathology manifesting as glaucoma.

The FEM predicts that temporal edge of the LC is displaced nasally and posteriorly during adduction tethering (Fig. 3). Nasal shifting of the ON central vascular trunk and LC has been observed during axial elongation of the myopic child’s eye, although without speculation as to its cause^65,66,68^. Displacement of the LC observed by optical coherence tomography^65,66,68^ corresponds to temporal peripapillary strain predicted by the FEM. The temporal region is the most common site of development of peripapillary atrophy, a feature that progresses from childhood^69^, is associated with deformation of that region during eye movement^37^, and has a typical morphology mirroring the stress and strain distributions evident in the simulations in Figs. 2, 4, and 5. This coincidence suggests that peripapillary atrophy may be induced by mechanical effects of accumulated horizontal eye movements.

The current FEM suggests that stresses near the ON junction when IOP is high and ICP is low, but without adduction tethering (7–21 kPa, Fig. 4) are within the range in several published FEM studies of the effect of IOP, ranging from 6.7 kPa^70^ to 22–30 kPa^71^ to as much as 213 kPa^72^. Figure 4 shows that ON tethering in adduction imposes higher stresses on the disc, retrolaminar ON, and peripapillary sclera than does markedly elevated IOP, even when augmented by low ICP to create a markedly abnormal translaminar pressure differential that roughly adds to the effect of adduction tethering.

While elevated IOP is unequivocally the cause of ON damage in narrow-angle and other secondary forms of high pressure glaucoma, the absence of abnormally elevated IOP in many cases of primary open angle glaucoma has given rise to the term “normal tension glaucoma” (NTG)^73,74^. Examination by MRI during adduction tethering demonstrates the ON to elongate less, and thus be stiffer than normal, in NTG^15^. The current FEM suggests that ON stiffness is a key determinant of optic disc stress. Figure 5 demonstrates that stress on the ON’s junction with the eye varies significantly with ON stiffness. In case B with “stiff” ON connective tissue (Table 1), stresses in visually critical posterior ocular regions are particularly high and increase further with IOP or translaminar pressure elevation (Fig. 5, lower right). As illustrated in Fig. 5 lower right, this IOP elevation can be considered to represent hoop-stress^75^ that is concentrated in the temporal optic disc and peripapillary region.

The current FEM inevitably has limitations. For simplification, the FEM assumed a fixed ocular rotational center, although the globe is actually supported by deformable connective tissue^76^ and fat that permit some globe translation during rotation^77–79^. The forcing function for the FEM was assumed to be a distributed rotational force exerted on the anterior sclera, while in actuality the balance of more focally applied medial and lateral rectus muscle forces rotate the eye. Since the real eye normally translates medially, and the glaucomatous eye also translates posteriorly in adduction^77–79^, adduction slightly larger than 6° would probably be required to produce the adduction tethering simulated here. However, since the physiologic range of adduction exceeds 40° in humans^80^, the current FEM operates far below the maximum adduction tethering that humans experience during everyday activities^2,9,80,81^. Employing an anatomically complete ocular suspensory system, including realistic connective tissues and extraocular muscles, will make the model more comparable to human the MRI studies^3,37^; however such a model would be considerably more complex and computationally intensive.

Of course, larger eye rotations associated with adduction tethering of the ON are brief transients that are not maintained continuously as is the case for IOP, and so may reduce their biological impact even though their maximal values may be one to two orders of magnitude greater than those associated with IOP. Perhaps offsetting this consideration, however, is that adduction tethering is likely to involve substantially higher transient stresses and strains that simulated in the current quasi-static model. Material properties employed in this quasi-static FEM were characterized during slow uniaxial tensile loading; viscoelastic properties^57^ are necessary to understand behavior during dynamic eye movements. The vestibulo-ocular reflex, an automatically generated ocular counter rotation during head rotation, is coordinated with large saccades during head movement, typically involving an average eye movement of about 30°^2,9,81^ and around 400°/s^82^ velocity, with accelerations often exceeding^83^ 6000°/s^2^. Viscous tissue properties would be expected to result in much higher transient stresses and strains on the eye during the rapid accelerations and decelerations associated with saccadic eye movements. The current FEM assumed isotropic tissue properties, which may not ideally characterize behavior of peripapillary sclera^84–87^. For clarity of presentation, this FEM assumed an eye of average size and shape. Forthcoming studies will investigate the effects of observed variation in sizes of the eyeball and orbit, and will consider dynamic effects of rapid eye movements when viscoelastic tissue properties have been experimentally characterized.

## Conclusion

A FEM simulating incremental ocular adduction from 26 to 32° predicts that adduction beyond the initiation of ON tethering concentrates stress and strain on the temporal side of the optic disc and peripapillary sclera, the ON sheath junction with the sclera, and retrolaminar ON neural tissue. Some unfavorable combinations of tissue properties within the published ranges imposed higher stresses in these regions, and the least favorable combinations of tissue properties was predicted to stress the ON junction and peripapillary sclera more than extreme conditions of intraocular and intracranial pressure. These simulations support the concept that ON tethering in adduction could induce mechanical stresses that might contribute to ON damage.

## Methods

### Model geometry

**Figure 6:**
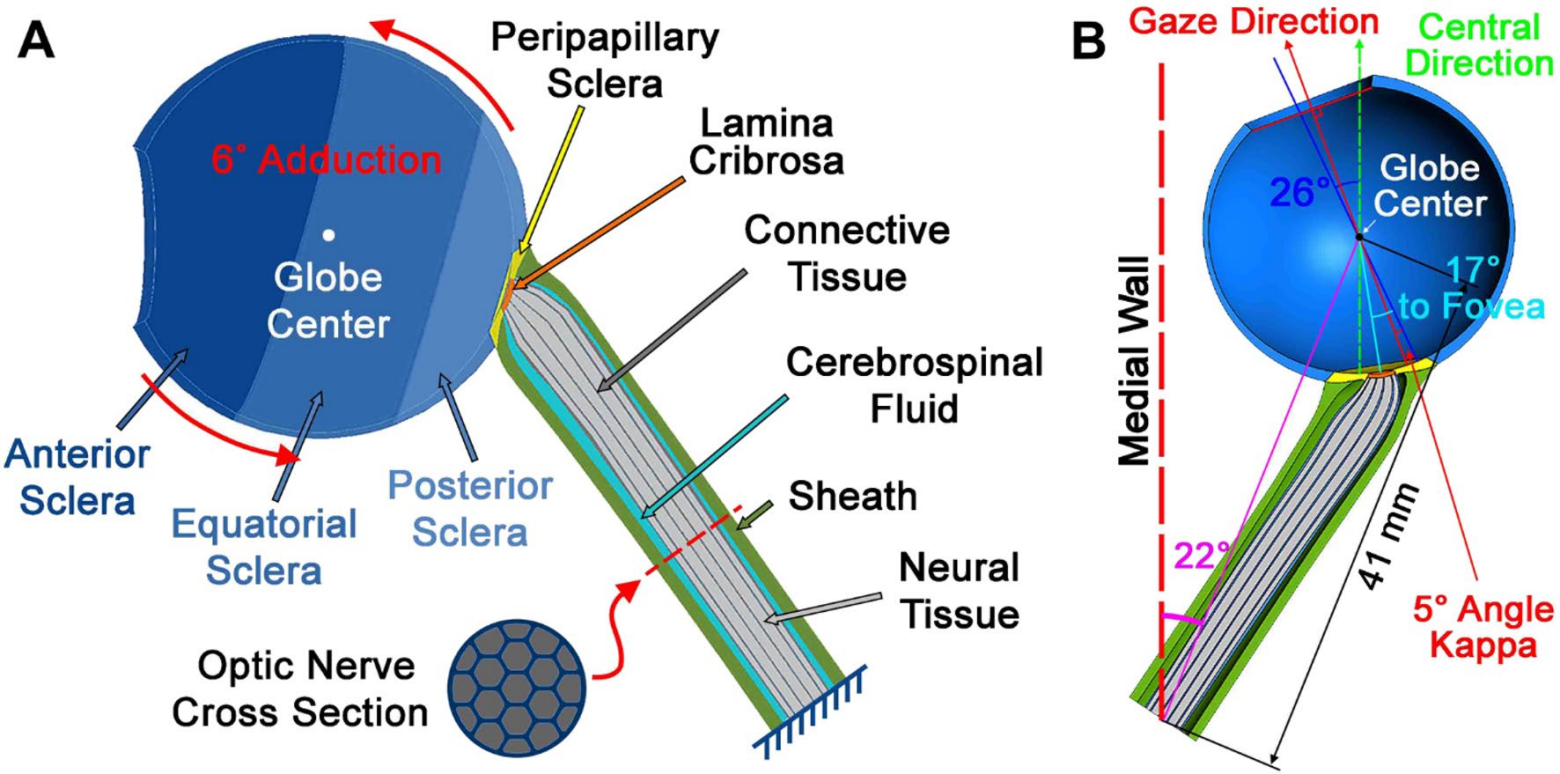
Model geometry. (**A**) Sclera is parsed into anterior, equatorial, posterior (blue) and peripapillary regions (yellow). The lamina cribrosa (orange) abuts the peripapillary sclera. The posterior lamina cribrosa is joined to the ON (gray), and the ON sheath (green) is joined to the peripapillary sclera anteriorly, and to the orbital apex posteriorly as boundary conditions. Cerebrospinal fluid (CSF) is shown in light blue. (**B**) Dimensions of the model.

A hemi-symmetric model was designed using SOLIDWORKS 2017 (Dassault Systèmes, Waltham, MA) for the initial condition 26° adduction at which the average ON first exhausts slack and thus becomes straightened^1,3^. The globe was assumed a 24 mm diameter sphere^88^, with scleral thickness ranging from 0.4 mm at the equator, increasing to 1 mm at the posterior pole, but thinner around the scleral canal^89,90^. The sclera was parameterized in four regions: peripapillary within 4 mm from the optic disc center^91,92^, and the remainder divided into anterior, equatorial, and posterior regions^43^. The model incorporated a 5° angle kappa^93^ and 17° angle between fovea and optic disc (Fig. 6). The lamina cribrosa (LC)^94,95^ was simplified as having a curved shape 1.8 mm in diameter and 0.3 mm thick^71,96,97^. MRI with T2 fast spin echo sequences and surface coils was performed as published^2,98–101^ to measure anatomical dimensions in 22 healthy subjects showing 41 mm mean distance between globe center and orbital apex, and a 22° mean angle between a line connecting those points and the medial orbital wall. We performed MRI, and all other procedures, in accordance with relevant guidelines and regulations after obtaining written informed consent from all subjects according to a protocol approved in advance by the University of California, Los Angeles Medical Institutional Review Board (IRB) and conforming with the Declaration of Helsinki.

**Figure 7:**
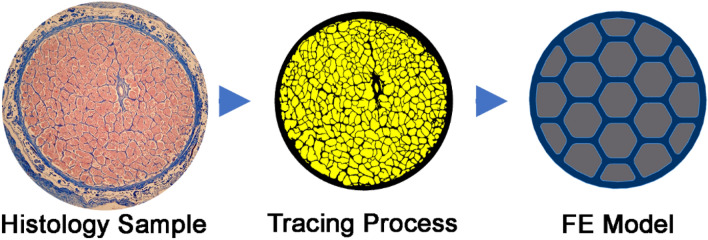
Model simplification of human optic nerve (ON). Transverse histological section of 57 year old human ON was chosen to calculate the proportion portion of connective versus neural tissue. In this 10 μm thick histological section stained with Mason trichrome at left, neural tissue (pink) was segmented from connective tissue (blue) as outlined as in the middle tracing. After assigning a 9:16 area ratio of connective to neural tissue, the ON was simplified as the honeycomb structure at right for modeling.

Microscopy has demonstrated that the ON pia is tightly coupled to a dense internal matrix of connective tissue (Fig. 7) intermingled with nerve axons^102^. Specimens examined here were legally obtained at autopsy and do not constitute human subject research requiring IRB review. As described previously by Karim et al., a human orbit (57 year old Caucasian female) embedded whole in paraffin was serially sectioned at 10 μm thickness in the plane perpendicular to its long axis^102^. A transverse section selected 0.65 mm posterior to the globe was processed with the Masson trichrome method that stains collagen blue and neural tissue purple. The specimen was photographed using a Nikon digital camera using a Nikon Eclipse microscope with 10 × objective. As described by Karim et al., colorimetric selection in Adobe Photoshop was employed to select the collagenous connective tissue in the ON for quantitation on a pixel basis with the overall dimension of the ON cross section^102^. On this basis, the ON was modeled as a honeycomb structure with intrinsic connective and neural tissues intermixed in histologically observed 9:16 proportions (Fig. 7). Also, we incorporated observed gradual tapering of ON diameter from 4 mm anteriorly to 3.5 mm posteriorly^102^. Thicknesses of the ON sheath and cerebrospinal fluid (CSF) layer were set to 0.74 mm and 0.59 mm, respectively, as measured in living humans by MRI^1^. The CSF layer was subjected to intracranial pressure (ICP).

### Implementing material properties

The model was simulated using ABAQUS 2020 (Dassault Systèmes, Waltham, MA), employing hyperelastic tissue properties fitted as reduced polynomial strain energy shown in Eq. (1) for each tissue as empirically described^43^. Equation (1) follows the instruction described in 6.13 Analysis User’s Guide. This function is given by

1 $$U = \sum\limits_{i = 1}^{N} {C_{i0} (\overline{I}_{1} - 3)^{i} + } \sum\limits_{i = 1}^{N} {\frac{1}{{D_{i} }}\left( {J^{el} - 1} \right)^{2i} }$$

where *U* is the strain energy per unit of reference volume, $\overline{I}_{1}$ is the first deviatoric strain invariant, and *N*, *C*_*i*0_ and *Di* are material parameters. We considered hyperelastic stress–strain curves at the 5th percentile to represent “compliant” behavior, and 95th percentile to represent “stiff” behavior; this is informal shorthand for published hyperelastic functions that were actually implemented^43^. Stiff, average, and compliant tissue properties are defined in Table 1. We employed published linear properties of brain for ON neural tissue (Table 1)^103^, and published nonlinear properties of the LC (Table 1)^104^.

The ON is a composite of soft axon bundles in a matrix of stiff connective tissue at a scale prohibiting individual measurement of each component’s tensile properties. The elastic modulus *E*_*Connective*_ for intrinsic connective tissue was therefore computed from the general rule of mixtures for a composite having the measured portions (Eq. 2). The general rule of mixtures was applied to estimate elastic modulus of connective tissue embedded with ON neural tissue:

2 $$E_{ON} = fE_{Connective} + (1 - f)E_{Neural}$$

where $f = \frac{{V_{Connective} }}{{V_{Connective} + V_{Neural} }}$ is the volume fraction of connective tissue, *E*_*Connective*_ is the ON connective tissue elastic modulus and *E*_*Neural*_ is the neural tissue modulus. The volume ratio of neural and connective tissue is explained in Fig. 7. We employed measured tensile elastic modulus (*E*_*ON*_) of the entire human ON^43^, and that of neural tissue published for brain^103^, allowing computation of *E*_*Connective*_ from Eq. (2). The von Mises criterion, a common yield criterion for ductile materials, was employed^105^.

### Boundary conditions

Electron microscopy reveals that the posterior arachnoid trabeculae coupling the ON to its sheath are thicker near the orbital apex than anteriorly^106^. Furthermore, we dissected unfixed human orbits (obtained from cadavers legally from anatomical donations) and found that both the ON and its sheath are rigidly fixed in the orbital apex, constituting their fixed boundary. The posterior end of ON and sheath were set to fixed condition. The eye’s rotational axis was fixed its center. Hemi-symmetry about the horizontal was assumed. Adduction was implemented using a forcing function to impose a uniform rotation on the anterior sclera.

### Input loading

From the 26° adducted initial configuration at which the ON first straightens, the forcing function imposed 6° further adduction. Unless otherwise stated, normal IOP (15 mmHg) and normal ICP (10 mmHg) were applied to the vitreous body and CSF layer, respectively. Pretension in the ON when sinuous at angles less than those inducing tethering was assumed zero^1,3^; greater pretension at that initial angle would increase the tissue loadings simulated here.

### Mesh

**Figure 8:**
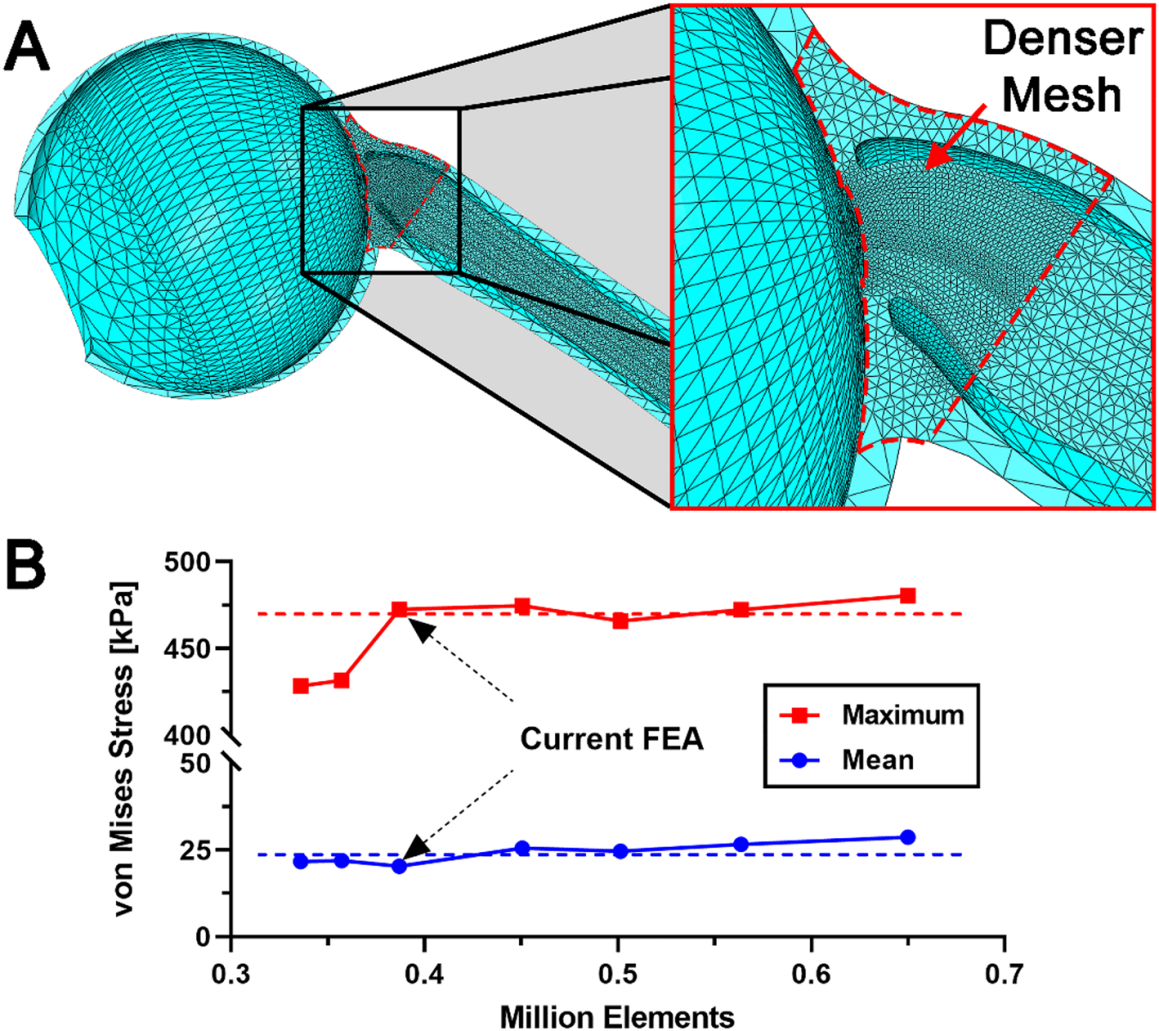
(**A**) Local mesh size was adjusted according to regional relevance, thus finest around the optic nerve head. (**B**) Mean and maximum values of von Mises stresses of all elements in the model were stable throughout all element numbers evaluated. Dotted lines indicate means.

The model was meshed using 386,983 10-node quadratic tetrahedral elements with sizes variably assigned depending on the region of interest (Fig. 8A) and confirmed by convergence testing (Fig. 8B). Since the globe-ON junction was of greatest interest, mesh density was incrementally evaluated here. Other regions, including anterior and posterior portion sclera, were coarsely meshed. For the comparison among individual cases, 12–16 contiguous elements in regions of interest were averaged to quantify local representative stress and strain.

## Data Availability

This is a primarily a theoretical paper. Data underlying the model are all previously published and referenced to the text, except for that illustrated in Fig. 7, which is its own original data.

## Code availability

The model structure is implemented using SolidWorks, and may be found at: https://zenodo.org/record/4287712#.YjEIV3rMIuU. The Abaqus input files for all cases are available at: https://zenodo.org/record/4976263#.YjEIl3rMIuU.
